# Preoperative Multistrain Probiotic Supplementation Does Not Affect Body Weight Changes or Cardiometabolic Risk Factors in Bariatrics: Randomized, Double-Blind, Placebo-Controlled Clinical Trial

**DOI:** 10.3390/nu16132055

**Published:** 2024-06-27

**Authors:** Marta Potrykus, Sylwia Czaja-Stolc, Marta Stankiewicz, Michał Szymański, Igor Łoniewski, Łukasz Kaska, Monika Proczko-Stepaniak

**Affiliations:** 1 Department of Oncological, Transplant, and General Surgery, Medical University of Gdansk, 80-211 Gdańsk, Poland; martapotrykus@gumed.edu.pl (M.P.); szymanski@gumed.edu.pl (M.S.); monika.proczko-stepaniak@gumed.edu.pl (M.P.-S.); 2 Department of Clinical Nutrition and Dietetics, Medical University of Gdansk, 80-211 Gdańsk, Poland; marta.stankiewicz@gumed.edu.pl; 3 Sanprobi sp. z o.o. sp. k., 70-535 Szczecin, Poland; sanprobi@sanprobi.pl; 4 Department of Biochemical Science, Pomeranian Medical University in Szczecin, 71-460 Szczecin, Poland; 5 Independent Public Health Care Center of the Ministry of Internal Affairs and Administration, 80-210 Gdańsk, Poland; lukasz.kaska@wp.pl

**Keywords:** probiotics, intestinal microbiota, bariatric surgery, obesity, metabolic disorders, weight loss

## Abstract

Emerging evidence suggests that microbiota plays a crucial role in the development, progression, and therapeutic options in obesity and its comorbidities. This study assessed preoperative probiotic therapy’s impact on bariatric treatment outcomes. A 12-week randomized, double-blind, placebo-controlled trial with 48 patients undergoing bariatric surgery was conducted. Participants received probiotics—Sanprobi Barrier—which contained nine strains of bacteria: *Bifidobacterium bifidum* W23, *Bifidobacterium lactis* W51 and W52, *Lactobacillus acidophilus* W37, *Levilactobacillus brevis* W63, *Lacticaseibacillus casei* W56, *Ligilactobacillus salivarius* W24, *Lactococcus lactis* W19, and *Lactococcus lactis* W58. Primary outcomes included excess body weight loss, body weight loss, and excess body mass index loss, with secondary objectives focusing on metabolic profiles. Surgical treatment of obesity significantly improved anthropometric and metabolic parameters. No significant differences were observed in primary outcomes or in secondary outcomes between groups at any time point post-surgery. Preoperative probiotics administration did not affect clinical outcomes 1, 3, or 6 months following bariatric surgery.

## 1. Introduction

Obesity is a disorder characterized by the accumulation of an excessive amount of body fat, leading to detrimental health consequences [1]. According to the latest data from the World Health Organization (WHO), in 2022, every eighth person worldwide suffered from obesity [2]. With the consistent rise in mortality and morbidity rates in individuals with excess body weight, gaining a comprehensive understanding of the mechanisms underlying prolonged and excessive overeating is of paramount importance [2] In 2019, an elevated body mass index (BMI) caused approximately 5 million deaths attributed to noncommunicable diseases (NCDs), including cardiovascular diseases, diabetes, cancer, neurological disorders, chronic respiratory diseases, neurological disorders, and digestive disorders [3].

Although bariatric surgery (BS) has shown remarkable efficacy in treating obesity and its comorbidities, there remains a subset of patients who fail to achieve the desired outcomes [4]. Obesity is a serious healthcare challenge due to its multifactorial nature.

Genetic predisposition, lifestyle factors, and dietary habits are recognized as leading causes of obesity, yet an emerging focus has been placed on the gut microbiota’s influence [5]. The gut microbiota in individuals with obesity significantly differs from that observed in individuals with normal body weight. People with excess body weight tend to show reduced bacterial diversity within the gut microbiome, often with an increased ratio of Bacillota (Firmicutes) to Bacteroidota (Bacteroidetes) [6]. Studies suggest that a 20% increase in Firmicutes and a proportional decrease in Bacteroidetes may result in an additional intake of approximately 150 kilocalories per day [7]. The intestinal microbiota contributes to increasing energy harvest by producing short-chain fatty acids (SCFA) and simple sugars through the breakdown of polysaccharides. Microorganisms stimulate higher nutrient absorption by enhancing blood vessel density in the intestine [8]. Notably, the dominance of specific bacteria associated with obesity inhibits fasting-induced adipose tissue factor (FIAF). This inhibition increases fat accumulation by diminishing fat oxidation in the liver and muscles [9]. Moreover, intestinal microbiota has been shown to reduce the secretion of intestinal hormones such as peptide YY (PYY) and glucagon-like peptide-1 (GLP-1). These hormones play a pivotal role in regulating satiety, and their decreased secretion may lead to heightened food consumption [10]. Probiotic supplementation before BS may influence postoperative outcomes through various mechanisms. Probiotic supplementation helps maintain a healthy balance of gut bacteria by promoting the growth of beneficial strains that compete with pathogenic bacteria for nutrients and colonization niches, producing substances called bacteriocins that inhibit the growth of detrimental microorganisms [11]. Probiotics regulate cytokine levels by enhancing anti-inflammatory cytokines while diminishing pro-inflammatory ones [12]. Additionally, probiotics strengthen the integrity and functionality of the intestinal barrier, thereby preventing the translocation of harmful substances from the gut epithelium into the bloodstream, which may alleviate adipose tissue inflammation associated with obesity [13]. Overall, preoperative probiotics may create a healthier gut environment, modulate the immune response, help alleviate the inflammatory response caused by surgery, and enhance the effect of bariatric treatment.

Conclusions drawn from studies investigating the impact of probiotic therapy post-bariatric surgery are inconsistent. Some suggest potential benefits, such as improvements in weight loss and metabolic state, while others indicate inconspicuous clinical effects. The variability in findings provoked thoughts on alternative approaches to supporting the intestinal microbiome to enhance the effectiveness of bariatric treatment. To the best of our current knowledge, there is a lack of scientific literature concerning probiotic therapy before surgical obesity treatment. Therefore, we have decided to examine whether implementing probiotic therapy preoperatively could enhance the outcomes of bariatric treatment. The primary aim of this study was to assess the impact of probiotic therapy on weight loss, with secondary objectives focused on evaluating metabolic profiles.

## 2. Materials and Methods

### 2.1. Study Design

The study was designed as a randomized, double-blind, placebo-controlled clinical trial with a 12-week probiotics intervention period. Participants were randomly divided into two research arms—the probiotic group receiving multistrain probiotics and the placebo group. Allocation to groups was performed at a 1:1 ratio using an Microsoft Excel version 2019 random number generator. The study was unblinded after the statistical analysis. The project was conducted between August 2021 and April 2023 at the University Clinical Center (UCC) in Gdańsk (Poland). The protocol was approved by the Independent Bioethics Committee for Scientific Research at the Medical University of Gdańsk (No. NKNNB/447/2021) in accordance with the Declaration of Helsinki. The study was registered with clinical trials under the identifier NCT05407090.

This study is the first to evaluate the effect of preoperative probiotic supplementation; hence there are no previous studies on which we could base our sample size calculation. Based on data from Sanchez et al. [14] (weight reduction due to probiotics in patients with obesity), assuming α = 0.05 and power = 80%, the initial calculation resulted in 20 patients per group. However, considering protocols variations and potential patient dropouts to ensure the study’s validity and reliability, we decided to include 40 patients per group, which gives a total of 80 participants. However, during the study, it was noted that the attrition rate among patients exceeded the anticipated rate of 10–15%, reaching levels of 20–30%. Consequently, the study’s recruited patient cohort was increased to 110 individuals.

### 2.2. Participants

One hundred and ten patients qualified for BS were enrolled in the study after screening interviews. The patients were qualified for Laparoscopic Sleeve Gastrectomy (LSG) or One Anastomosis Gastric Bypass (OAGB). The inclusion criteria included eligibility for BS based on International Federation for the Study of Obesity (IFSO) guidelines [15], Caucasian race, and age over 18. Patients were excluded from the study if they met any of the exclusion criteria: allergy/intolerance to any of the ingredients of the preparations; inflammatory bowel diseases; current antibiotic therapy; immunosuppression; biological treatment; long-term antibiotic therapy; probiotic therapy in the 1 month before study enrollment; neurodegenerative diseases, and antipsychotic drugs. Operational treatments were performed in accordance with the Enhanced Recovery After Bariatric Surgery (ERABS) protocol [16].

### 2.3. Study Protocol

Baseline and follow-up evaluations were performed in the University Clinical Center (UCC) in Gdańsk. Both research arms received identical medical care during the study period. Anthropometric measurements and blood samples were collected from the patients during each visit. After 12 weeks of supplementation, patients underwent either LSG or OAGB surgery. Subsequent follow-up visits were scheduled at 1 month (1 M PostOP), 3 months (3 M PostOP), and 6 months post-surgery (6 M PostOP). The patients followed a standardized diet under the supervision of a qualified dietitian. Upon recruitment, participants were provided with a balanced diet plan, which included a calorie deficit of 500–1000 kilocalories. Nutrient requirements were set at 25–30% for protein, 25–30% for fats, and 45–55% for carbohydrates of total daily energy intake, while mineral and vitamin requirements were based on the dietary standards for the Polish population [17]. Post-surgery, the dietary plan remained consistent, with 5–6 high-calorie-density meals per day. Fiber intake ranged from 10–30 g or 15 g/800–1000 kcal, depending on individual tolerance and time elapsed since the operation. All patients were prescribed vitamin supplements (WLS FitForMe, Rotterdam, The Netherlands).

### 2.4. Probiotic Intervention

The study product was a probiotic mixture contained nine strains of bacteria: Bifidobacterium bifidum W23, Bifidobacterium lactis W51 and W52, Lactobacillus acidophilus W37, Levilactobacillus brevis W63, Lacticaseibacillus casei W56, Ligilactobacillus salivarius W24, Lactococcus lactis W19, and Lactococcus lactis W58 in daily dose 2 × 10^9^ CFU. This product is commercially sold in Poland under the name of Sanprobi Barrier (Sanprobi sp. z o. o. sp. k., Szczecin, Poland) and has been authorized by the appropriate health authorities regarding its composition and recommended dosage. Identical-looking placebo capsules contained maize starch and maltodextrin of maize origin. Patients were instructed to store the products in the refrigerator and take four capsules daily with meals (two capsules in the morning and two capsules in the evening) for 12 weeks. Patients were asked to continue supplementation until the last meal before the operation, within 24 h before the surgery.

### 2.5. Nutritional Status Assessment

Patients had their height and weight measured, and their BMI was calculated. The evaluation of the results of surgical treatment of obesity was based on the percentage of excess body weight loss (%EWL), the percentage of body weight loss (%WL), and the percentage of excess BMI loss (%EBMIL) [18]. In calculating %EWL, the ideal body weight was determined using the Deitel and Greenstein formula from 2003 [19]. The initial weight and BMI were measured during the first visit to the clinic.

### 2.6. Metabolic Parameters

All blood samples were collected following a 12 h fasting period and analyzed by a certified laboratory UCC in Gdańsk. The following parameters were determined: lipid panel, liver enzymes, anemia diagnostics, folic acid, vitamin B_12_, glucose metabolism markers, and C-reactive protein (CRP). Insulin resistance was calculated using the homeostatic model assessment of insulin resistance (HOMA-IR) with the formula: HOMA-IR = [fasting blood glucose (mg/dL) × fasting insulin (mU/L)]/405.

### 2.7. Postoperative Complications

All postoperative complications were classified according to the 30 day postoperative Clavien–Dindo classification, which enables a standardized assessment of surgical complications. The complications were divided into five main grades (I–V), with grade I including the least severe complications that do not require intervention and grade V indicating the patient’s death [20].

### 2.8. Statistical Methods

Statistical analysis involved using different methods based on the type and distribution of the data. For normally distributed data, measures like means and standard deviations (SDs) were employed, while non-normally distributed data were analyzed using medians and interquartile ranges (IQRs). The normality of the data was examined using the Shapiro–Wilk test and visually inspected through histograms. Categorical data were compared using contingency tables, chi-squared tests, or Fisher’s exact test for limited observations (<5). Comparisons for normally distributed continuous data were done with *t*-tests or Welch’s correction for unequal variances. To analyze the differences in continuous variables between treatment groups across multiple time points, repeated measures mixed ANOVA was conducted, followed by post hoc tests. Statistical significance was considered at a *p*-value of less than 0.05 for all two-sided tests. The statistical analyses and graphical representations were conducted using GraphPad Prism 10.2.1 and JASP statistical software version 0.18.1 and the Python version 3.9.16.

## 3. Results

### 3.1. Flow of Trial Participants and Comparison between Arms

Out of 174 patients interviewed for eligibility, 64 did not fulfill the criteria or declined participation. Given the increasing societal awareness of the importance of the intestinal microbiome, many prospective study candidates were already consuming probiotics, thus meeting one of the exclusion criteria. Consequently, 110 individuals were randomly assigned to either the placebo (*n* = 55) or probiotic (*n* = 55) group. Forty-eight patients (43.6%) completed the protocol and attended all four scheduled visits. To accomplish the 12-week intervention, patients were recruited 3 months before the surgery. Due to the individualized treatment approach, the time between recruitment and surgery, and consequently the duration of the intervention varied among patients. Therefore, only patients who underwent supplementation for at least 6 weeks were included in the study. Considering patients’ treatment discontinuation (*n* = 19), withdrawals from the study (*n* = 6), supplementation durations less than 4 weeks (*n* = 1), non-adherence to the study protocol (*n* = 13), or absence at one of the follow-up visits (*n* = 13), a total of 48 individuals were included in the statistical analysis. Therefore, the study comprised 22 individuals in the probiotics and 26 in the placebo group.

**Figure 1:**
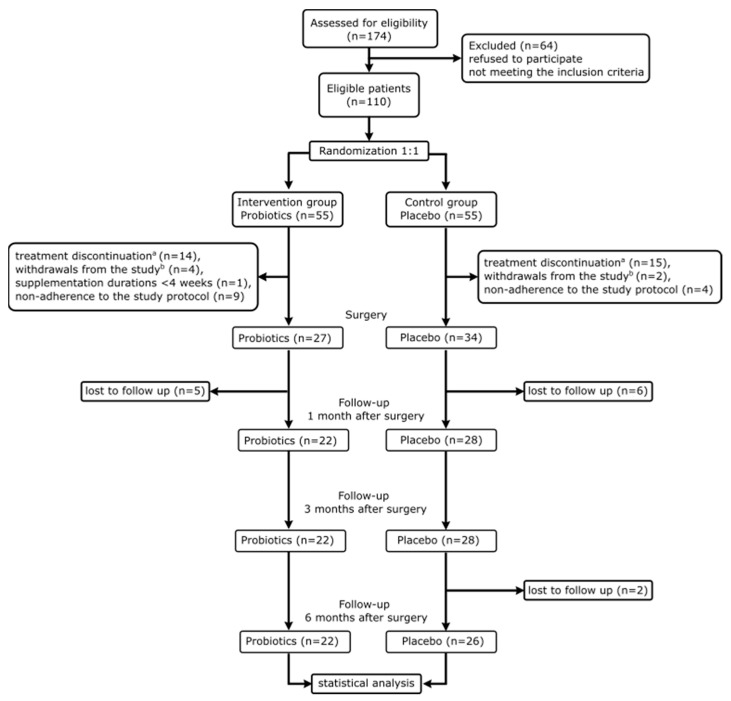
Study flow. ^a^ Treatment pathway was interrupted and participants did not proceed to surgery; ^b^ participants remained in the treatment cycle but opted out of participating in the research study. The absence of patients at the 1-month post-operation (1 M PostOP) visit was not due to non-compliance with the protocol but because some patients had a different treatment path that did not include a visit one month after surgery. To standardize the population, they were excluded from the statistical analysis in this publication.

One patient withdrew from the study due to abdominal pain and nausea after starting supplementation. After unblinding the study, it was revealed that he/she was taking a placebo. Another patient (with probiotics) reported a change in stool consistency after beginning supplementation, but the symptoms resolved within a week, and the patient remained in the study. This could have been a reaction to adaptation following the initiation of probiotic supplementation. According to Almutairi et al. [21], maltodextrin contained in the placebo may affect human gut physiology, but since both the placebo and the probiotics contained this substance, it should not have influenced the outcomes. The study flow is presented in Figure 1.

All baseline characteristics were similar in both study arms, including age (42.2 ± 11.6 vs. 41.0 ± 11.2 years), initial BMI (43.6 ± 4.4 vs. 43.2 ± 7.1 kg/m^2^), duration of supplementation (11.5 ± 1.9 vs. 11.5 ± 2 weeks) for the placebo and probiotics groups, respectively (Table 1). None of the assessed parameters showed significant differences between the groups before the intervention, except for folic acid, which was higher in the probiotic group (5.8 ± 1.6 vs. 7.2 ± 2.6; *p* = 0.028) (Table 2).

### 3.2. Primary Outcomes

**Figure 2:**
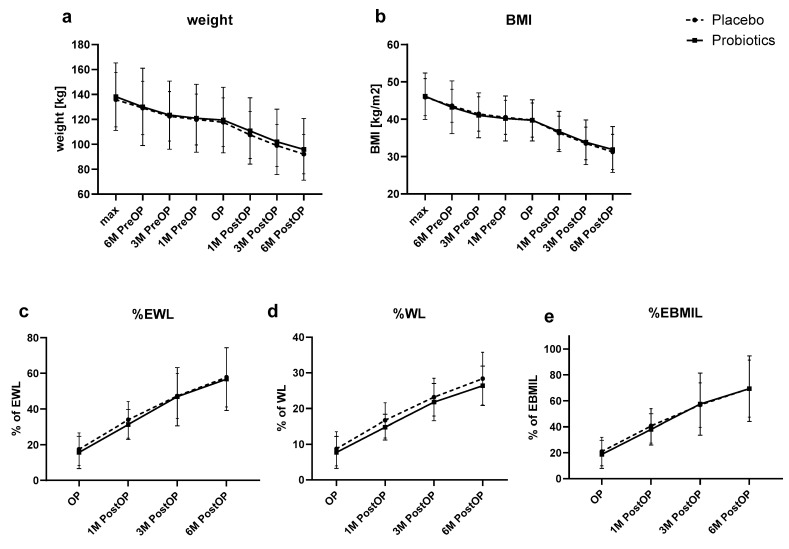
Comparison of primary outcomes between two groups at various time points. BMI—body mass index; %EWL—percentage of excess weight loss, %WL—percentage of weight loss; %EBMIL—percentage of excess body mass index loss. (**a**) Comparison of Weight Over Time Between Groups; (**b**) Comparison of BMI Over Time Between Groups; (**c**) Comparison of %EWL Over Time Between Groups; (**d**) Comparison of %WL Over Time Between Groups; (**e**) Comparison of %EBMIL Over Time Between Groups.

Patients’ weights and BMIs were significantly improved at 1 M, 3 M, and 6 M post-op compared to baseline in both arms (*p* < 0.001 for all), with no difference between the groups. Similarly, no significant differences between the groups were observed in %WL, %EWL, and %EBMIL at any of the follow-up visits. Results are presented both in Table 3 and Figure 2.

### 3.3. Secondary Outcomes

In this section, we present the secondary outcomes of the study. The data in Table 4 and Table 5 provide detailed results on these outcomes.

#### 3.3.1. Glycemic Parameters

Table 4 illustrates that all glycemic parameters—glucose, insulin, HbA1c%, HbA1c, and HOMA-IR—were significantly improved at 1 M, 3 M, and 6 M PostOP compared to baseline in both arms (*p* < 0.001 for all), with no difference between the groups.

#### 3.3.2. Lipid Profile

As shown in Table 4, triglycerides were significantly improved at 1 M, 3 M, and 6 M PostOP compared to the initial value (*p* < 0.001 for all). High-density lipoprotein (HDL) significantly decreased in 1 M PostOP but then increased at 6 M PostOP compared to baseline (*p* < 0.001 for both). There was no improvement in low-density lipoprotein (LDL) concentration after BS. The concentration of total cholesterol decreased compared to the baseline value at 1 (*p* < 0.001) and 3 M PostOP (*p* = 0.01), but the effect did not persist at the 6-month post-surgery check-in. No statistically significant differences were observed in the concentrations of triglycerides, HDL, LDL, and total cholesterol between the groups at any time point.

#### 3.3.3. Liver Enzymes

Lactate dehydrogenase (LDH) and gamma-glutamyl transferase (GGT) were significantly reduced in all time points in comparison to baseline (*p* < 0.013 for all time points for both parameters). The concentration of alkaline phosphatase (ALP) improved compared to the baseline value at 1 (*p* < 0.009), but the effect did not persist over time. There was no change in aspartate aminotransferase (AST) after surgery compared to initial value. No differences were noted between the groups in the concentrations of any liver enzymes at any time point (Table 4).

#### 3.3.4. Iron Parameters

As indicated in Table 4, iron was increased at 6 M PostOP in comparison to baseline (*p* < 0.008) but with no differences at other time points. There was no change in hemoglobin concentration after BS. No statistically significant differences were observed in the concentrations of iron and hemoglobin between the groups.

#### 3.3.5. Vitamins

Foliate and vit. B_12_ were significantly increased in all time points in comparison to baseline (*p* < 0.036 for all time points for both parameters). Vitamin D was increased 6 months post-op in comparison to baseline (*p* < 0.019). No significant differences were observed in the concentrations of vitamins between the groups. The comparison of concentrations of all examined parameters is presented in Table 4.

#### 3.3.6. Postoperative Complications

During the 30-day postoperative period, complications classified according to the Clavien–Dindo scale occurred in two patients (7.7%) in the placebo group and in three patients (13.6%) in the probiotics group. In the probiotics group, single cases of Grade I, IIIA, and IIIB complications were noted, while in the placebo group, only Grade II complications occurred (7.7%). No Grade IVA, IVB, or V complications were observed in either group. There were no statistically significant differences in the incidence of complications between the groups (Table 5).

## 4. Discussion

In the presented study, we have observed that probiotic supplementation before BS did not affect body weight, BMI, or any of the recommended tools for assessing the anthropometric effects of bariatric surgery, i.e., %WL, %EWL, %EBMIL at any time point, nor did it improve the metabolic profile of patients at any time point after surgery. To the best of our knowledge, this is the first randomized, double-blind, placebo-controlled clinical trial assessing the impact of probiotic therapy on the effects of surgical treatment of obesity in which all supplementation occurs before surgery.

In a meta-analysis conducted by Mohamed Aziz Daghmouri et al., parameters corresponding to our main goals, such as BMI change, absolute and percentage weight change from baseline, %EWL, fat mass change, and waist circumference change did not differ between the placebo and probiotics groups 3 months after surgery [22]. In another meta-analysis, Wang et al. observed that individuals supplementing with probiotics exhibited lower body weight three months after BS compared to those receiving a placebo. However, other anthropometric measurements, such as %EWL, BMI, or waist circumference, showed no differences between the two groups [23].

The gut microbiome plays a significant role in glucose metabolism, liver function, lipid profile, vitamins levels, and iron metabolism. Microbiota influences glucose metabolism through modulation of insulin sensitivity and secretion, as well as impacting glucose absorption and utilization [24]. The link between probiotics and the liver’s function involves the direct transport of gut-derived products to the liver via the portal vein. Metabolites from the gut microbiota can directly affect the liver’s functions, influencing its susceptibility to metabolical changes [25]. The gut microbiota may influence plasma cholesterol levels through bile acid metabolism and the production of dehydrogenase enzymes (ismA), which transform cholesterol into poorly absorbed sterol coprostanol, reducing serum total cholesterol levels [26]. The intestinal microbiome can influence iron metabolism by increasing the acidity of the environment, producing enzymes that convert ferric iron (Fe^3+)^ to ferrous iron (Fe^2+^), or by producing chelating substances that hinder its absorption [27,28]. Moreover, the interaction between host and microbiota metabolism affects vitamin levels [29].

Research on the effects of probiotic supplementation before BS is inconsistent. In a meta-analysis by Mohamed Aziz Daghmouri et al., insulin concentrations were lower in the probiotics group, with no differences in HbA1c levels [22]. Meanwhile, in a meta-analysis conducted by Wang et al., no differences were observed in any of the assessed glycemic parameters—glucose, insulin, HbA1c, HOMA-IR, and quantitative insulin sensitivity check index (QUICKI) [23]. In the work of Sherf-Dagan et al., a 6-month supplementation was carried out before BS in non-alcoholic fatty liver disease patients. It shows that the differences in AST, ALT, and GGT concentrations before 3, 6, and 12 months after the intervention did not differ between the probiotics and placebo groups [30]. Conversely, in the meta-analysis conducted by Wang et al., probiotics showed significant effects on regulating AST levels [23]. The meta-analysis conducted by Wang et al. revealed that while probiotic supplementation did not impact total cholesterol, LDL, or HDL cholesterol levels, there was a greater reduction in triglyceride levels among the probiotics group compared to the placebo 3 M postoperatively [23]. Daghmouri et al. revealed that no significant difference was found in HDL-cholesterol levels between the groups three months post-surgery. However, a significantly greater reduction in triglyceride and LDL levels was observed in the probiotic group compared to the placebo [22]. Regarding iron metabolism, studies indicate that probiotic therapy before BS does not significantly affect iron parameters such as ferritin and hemoglobin levels [23]. Some studies showed that probiotics supplementation increased the concentration of vitamin D in serum after surgery compared to the placebo group [31,32]. In our study, we did not observe any impact of multistrain probiotic supplementation on the metabolic profile after BS. However, it is worth noting that all the above-described publications referred to studies in which probiotic therapy was introduced after surgery. We did not find any other studies that examined the impact of probiotic therapy as a preparatory element before surgery on the outcomes of surgical treatment for obesity.

The multi-strain probiotic was used in this study because in earlier examinations, it demonstrated a positive impact on anthropometric and metabolic risk factors. It also enhanced the integrity of the gut barrier [33,34,35] and altered the effects of microbiota on biochemical, physiological, and immunological factors associated with obesity and inflammation [36]. A possible explanation for the lack of the expected favorable results in the group with probiotics is the fact that prehabilitation, as well as the surgery itself, have a significant impact on the host–microbiome metabolism [37]. Other studies in patients undergoing BS have also failed to demonstrate the efficacy of this multi-strain probiotic in reducing the severity of depressive symptoms, with the surgery itself leading to a significant reduction in both the probiotic and placebo groups [38]. The effect of probiotic therapy may be overshadowed by the substantial impact of prehabilitation and surgery on the microbiome, anthropometry, and metabolic status of patients. BS significantly affects gut microbiota [39]. Additionally, the perioperative preparation of the patient for surgery also contributes to the depletion of the intestinal microbiota. Surgical interventions disrupt the integrity of the intestinal barrier, potentially leading to increased bacterial translocation and systemic inflammation. Moreover, these interventions result in the reconstruction of the digestive tract and alterations in environmental conditions for microorganisms, which may cause significant changes in the local microbiome [40]. Consequently, this may decrease the effects of modifying the intestinal microbiota before surgery. Probiotic therapy appears to directly affect the patient’s metabolism during its administration. This implies that the timing of probiotic supplementation—before and after BS—may cause different effects. Supplementation before surgery focuses on optimizing patients’ health before BS, which may alleviate early postoperative gut dysbiosis caused by microbiome changes during surgery, reduce inflammation, improve metabolic profile, and promote recovery [41]. Postoperative probiotics therapy emphasizes maintaining a balanced microbiome after surgery, which can potentially directly influence patients’ weight, low-grade inflammation, and metabolic profile as well as mitigate gastrointestinal symptoms caused by the surgical intervention [32,42]. In addition, the effects of probiotics are strain-dependent; it is still not fully understood which strains, in what amounts, and at what time will be effective in modeling the intestinal microbiota to optimally influence the host’s metabolism and body weight. Currently, we have scientific evidence that the state of the intestinal microbiome is related to the development, progression, and therapeutic options in obesity and its consequences. Still, the full potential of this knowledge is not yet used in practice. The composition and activity of the gut microbiota vary individually, thus, there is no universal method of microbiota modification that would bring health benefits. Therefore, it is crucial to focus on performing thorough microbiota research in individual disease entities and to try to target microbiota modifications in narrow groups of patients. The strong point of the presented study is its design as a randomized, double-blind, and placebo-controlled research in which randomization was successful; both demogeographic data and assessed parameters in baseline did not differ between presented groups. One of the limitations of our study is the low number of patients. Despite recruiting the planned number of participants, less than 50% of patients qualified for statistical analysis, which reduced the power of the study. Therefore, it is possible that we did not detect existing differences between the groups. However, this sample size was sufficient to confirm the impact of the intervention on numerous anthropometric and biochemical parameters. It is likely that the effect of the probiotic is not sufficient to influence these parameters significantly. Therefore, caution should be exercised when interpreting the data and comparing it with the literature. Additionally, bariatric patients are a specific group whose diet, both before and after surgery, is low-calorie and possibly insufficient to ensure optimal nutrition for the microorganisms inhabiting the digestive tract. It would be valuable to repeat the presented study on a larger number of patients, but also with a changed methodology, where patients would receive both probiotics and prebiotics. Moreover, microbiota analysis would shed more light on the obtained results.

## 5. Conclusions

In conclusion, it seems that preoperative probiotic administration does not affect weight loss and clinically significant metabolic parameters in patients treated with BS. However, due to the limited population of participants, it is possible that we may underestimate the existing differences between the groups. Therefore, caution should be exercised in the interpretation and comparison of our findings with other literature. Additional high-quality randomized controlled trials will be necessary in the future to further explore the potential therapeutic effects of probiotics in patients with morbid obesity undergoing bariatric treatment.

## Figures and Tables

**Table 1:** Preintervention characteristic of groups.

	Placebo (*n* = 26)	Probiotics (*n* = 22)	*p*-Value
Sex (F/M) *n*	20/6	13/9	0.184
Age (years)	42.2 ± 11.6	41 ± 11.2	0.701
Duration of supplementation (weeks)	11.5 ± 1.9	11.5 ± 2	0.965 †
Max weight (kg)	135.9 ± 21.8	138.3 ± 27.1	0.733
Weight 6 M PreOP (kg)	129.2 ± 21.4	130.1 ± 31.0	0.907
Max BMI (kg/m^2^)	45.9 ± 5.0	46.2 ± 6.2	0.885
BMI 6 M PreOP (kg/m^2^)	43.6 ± 4.4	43.2 ± 7.1	0.825
Type of surgery (LSG/OAGB)	24/2	18/4	0.392 ‡
current-smokers *n* (%)	7 (26.9)	1 (4.5)	0.055 ‡
ever-smokers *n* (%)	9 (34.6)	9 (40.9)	0.584
DM1 *n* (%)	0	1 (4.5)	0.458 ‡
DM2 *n* (%)	6 (23.1)	6 (27.3)	0.738
HTN *n* (%)	14 (53.8)	12 (54.5)	0.961
DL *n* (%)	17 (65.4)	15 (68.2)	0.838
HT *n* (%)	9 (34.6)	7 (31.8)	0.838
fatty liver *n* (%)	21 (80.8)	19 (86.4)	0.604
OSAS (%)	17 (65.4)	17 (77.3)	0.367
Impaired fasting glucose *n* (%)	9 (34.6)	11 (0.5)	0.281

Abbreviations: BMI—body mass index; LSG—laparoscopic sleeve gastrectomy; F-female; M—male; OAGB—one anastomosis gastric bypass; DM1—diabetes mellitus type 1; DM2—diabetes mellitus type 2; HTN—hypertension; DL—dyslipidemia; HT—hypothyroidism; OSAS—obstructive sleep apnea syndrome; ‡—Fisher’s exact test; †—U Mann–Whitney test.

**Table 2:** Preintervention laboratory parameters.

	Placebo (*n* = 26)	Probiotics (*n* = 22)	*p*-Value
Vit. D (pg/mL)	49.9 ± 15.1	52.5 ± 15.5	0.562
Folic Acid (ng/mL)	5.8 ± 1.6	7.2 ± 2.6	0.028
Vit. B_12_ (pg/mL)	349.8 ± 93.6	349.4 ± 113.6	0.99
Iron (ug/dL)	77.1 ± 30.8	82.5 ± 37.9	0.59
Insulin (uU/mL)	20.9 ± 14.9	22 ± 18.1	0.827
LDH (U/L)	192.5 ± 41.8	196.2 ± 32.7	0.738
ALT (U/L)	37.6 ± 18.3	34.7 ± 16.8	0.573
AST (U/L)	24.8 ± 10.9	24.2 ± 10.6	0.867
GGT (U/L)	46.4 ± 31.2	36.8 ± 18.6	0.212
ALP (U/L)	84.6 ± 26.1	75 ± 18.4	0.154
TG (mg/dL)	147.8 ± 67	140.5 ± 70.1	0.715
HDL (mg/dL)	43.5 ± 8.6	47.1 ± 13.1	0.262
LDL (mg/dL)	117.8 ± 39.3	115.1 ± 27.8	0.787
Cholesterol (mg/dL)	187.7 ± 45.3	188.5 ± 34.9	0.942
HbA1c%	5.7 ± 0.7	6.1 ± 1.3	0.287
HbA1c (mmol/mol)	39.2 ± 7.2	42.7 ± 14.4	0.272
Glucose (mg/dL)	102.3 ± 16.4	112.9 ± 41.3	0.238
Hemoglobin (g/dL)	13.8 ± 1.5	14.2 ± 1.8	0.45
HOMA-IR	5.6 ± 5.5	6.4 ± 6.4	0.633

Abbreviations: Vit. D—vitamin D; Vit. B_12_—vitamin B_12_; LDH—lactate dehydrogenase; ALT—alanine aminotransferase; AST—aspartate aminotransferase; GGT—gamma-glutamyl transferase; ALP—alkaline phosphatase; TG—triglycerides; HDL—high-density lipoprotein; LDL—low-density lipoprotein; HbA1c%—glycated hemoglobin percentage; HbA1c—glycated hemoglobin; HOMA-IR—homeostatic model assessment for insulin resistance.

**Table 3:** Primary outcomes.

	Placebo (*n* = 26)	Probiotics (*n* = 22)	Between Subjects Effects (Group)	Within Subjects Effects (Time)	Within Subjects Effects (Time × Group)
Weight (kg)					
Max	135.9 ± 21.8	138.3 ± 27.1	0.749	<0.001	0.612
6 M PreOP	129.2 ± 21.4	130.1 ± 31			
3 M PreOP–PRO	122.5 ± 19.8	123.4 ± 27.4			
1 M PreOP	119.9 ± 20.4	120.9 ± 27.3			
OP	117.7 ± 19.5	119.5 ± 26.3			
1 M PostOP	107.5 ± 18.9	110.8 ± 26.6			
3 M PostOP	99.1 ± 16.9	102 ± 26.3			
6 M PostOP	92.1 ± 15.8	96 ± 24.7			
BMI (kg/m^2^)					
Max	45.9 ± 5	46.2 ± 6.2	0.966	<0.001	0.582
6 M PreOP	43.6 ± 4.4	43.2 ± 7.1			
3 M PreOP–PRO	41.4 ± 4.6	41.1 ± 6			
1 M PreOP	40.5 ± 4.6	40.2 ± 6			
OP	39.8 ± 4.6	39.7 ± 5.5			
1 M PostOP	36.3 ± 4.5	36.7 ± 5.4			
3 M PostOP	33.5 ± 4.4	33.8 ± 6			
6 M PostOP	31.2 ± 4.7	31.9 ± 6.1			
%WL					
OP	8.7 ± 4.8	7.7 ± 4.5	0.229	<0.001	0.739
1 M PostOP	16.7 ± 4.9	14.8 ± 3.6			
3 M PostOP	23.2 ± 5.3	21.8 ± 5.2			
6 M PostOP	30.4 ± 11.5	26.5 ± 8.8			
%EWL					
OP	17.5 ± 9.1	15.7 ± 9.1	0.636	<0.001	0.791
1 M PostOP	33.9 ± 10.2	31.3 ± 8.4			
3 M PostOP	47.3 ± 12.6	46.9 ± 16.3			
6 M PostOP	57.8 ± 16.7	56.8 ± 17.6			
%EBMIL					
OP	20.9 ± 11	18.8 ± 10.8	0.803	<0.001	0.7
1 M PostOP	40.7 ± 13.3	38 ± 12			
3 M PostOP	56.7 ± 17.1	57.4 ± 23.9			
6 M PostOP	72.6 ± 34.6	62 ± 40.4			

Abbreviations: BMI—body mass index; %WL—percentage of weight loss; %EWL—percentage of excess weight loss; %EBMIL—percentage of excess BMI loss.

**Table 4:** Comparison of Study Secondary Outcomes Between Groups.

	Baseline		1 M PostOP		3 M PostOP		6 M PostOP		Between Subjects Effects (Group)	Within Subjects Effects (Time)	Within Subjects Effects (Time × Group)
	Placebo *n* = 26	Probiotics *n* = 22	Placebo *n* = 26	Probiotics *n* = 22	Placebo *n* = 26	Probiotics *n* = 22	Placebo *n* = 26	Probiotics *n* = 22	*p*-Value	*p*-Value	*p*-Value
Vit. D (pg/mL)	49.9 ± 15.1	52.5 ± 15.5	51.5 ± 12.6	58.7 ± 18.5	52.4 ± 15.5	63.5 ± 18.6	57.8 ± 21.3	60.1 ± 18.2	0.132	0.018	0.276
Folic Acid (ng/mL)	5.8 ± 1.6	7.2 ± 2.6	8.7 ± 3.2	8.7 ± 2.9	8.9 ± 3.7	8.6 ± 3.3	11.8 ± 8.3	8.8 ± 4.1	0.517	0.002	0.097
Vit. B_12_ (pg/mL)	349.8 ± 93.6	349.4 ± 113.6	483.2 ± 169.8	472.3 ± 147.4	455.4 ± 202	422.1 ± 182.1	411 ± 142.6	397.6 ± 163.1	0.694	<0.001	0.866
Iron (ug/dL)	77.1 ± 30.8	82.5 ± 37.9	72.5 ± 20.9	72.4 ± 26.1	79.2 ± 25.5	86.7 ± 32.3	85.7 ± 27.1	92.8 ± 36.4	0.408	0.016	0.845
Insulin (uU/mL)	20.9 ± 14.9	22 ± 18.1	8.5 ± 3.8	13.5 ± 9.1	7.8 ± 3.4	9.3 ± 5.4	7.3 ± 2.4	9.3 ± 8	0.168	<0.001	0.527
LDH (U/L)	192.5 ± 41.8	196.2 ± 32.7	179.5 ± 38.1	175.7 ± 34.2	160.9 ± 46.8	176.7 ± 44.6	160.7 ± 33.6	163.2 ± 36.1	0.618	<0.001	0.35
ALT (U/L)	37.6 ± 18.3	34.7 ± 16.8	35.6 ± 18.7	44 ± 25.5	24.1 ± 23.1	27.5 ± 14.5	17.9 ± 7.4	20 ± 8.8	0.421	<0.001	0.34
AST (U/L)	24.8 ± 10.9	24.2 ± 10.6	21.3 ± 7.5	24.9 ± 8.8	22.2 ± 24.5	25.2 ± 20.7	17.7 ± 5.6	17.8 ± 6.7	0.536	0.072	0.795
GGT (Ul)	46.4 ± 31.2	36.8 ± 18.6	28.3 ± 21.1	28.2 ± 15.4	25.5 ± 18	28.7 ± 32	24.5 ± 23.3	34.3 ± 54.3	0.907	0.006	0.132
ALP (U/L)	84.6 ± 26.1	75 ± 18.4	72.1 ± 22.2	69.6 ± 15.9	74 ± 24.2	77.6 ± 22.7	79.7 ± 25.2	82.7 ± 25.5	0.811	0.001	0.065
TG (mg/dL)	147.8 ± 67	140.5 ± 70.1	111.6 ± 52	110.6 ± 29.8	105.1 ± 56.1	99.2 ± 27.4	99.6 ± 41.3	99.1 ± 39.9	0.756	<0.001	0.901
HDL (mg/dL)	43.5 ± 8.6	47.1 ± 13.1	35.8 ± 6.2	39.2 ± 8.1	40.8 ± 5.6	45.3 ± 8.7	47.5 ± 6.9	51.9 ± 10.3	0.061	<0.001	0.906
LDL (mg/dL)	117.8 ± 39.3	115.1 ± 27.8	109.2 ± 40.3	105.6 ± 29	112.5 ± 40.4	114.5 ± 29.3	112.5 ± 34.1	117.7 ± 36	0.981	0.194	0.697
Cholesterol (mg/dL)	187.7 ± 45.3	188.5 ± 34.9	160.7 ± 41.9	162.7 ± 34.6	168.6 ± 47.9	173.3 ± 33.4	169.5 ± 43.1	182 ± 41	0.613	<0.001	0.665
HbA1c%	5.7 ± 0.7	6.1 ± 1.3	5.2 ± 0.5	5.4 ± 0.8	5.3 ± 0.4	5.3 ± 0.7	5.3 ± 0.5	5.5 ± 1.1	0.301	<0.001	0.5
HbA1c (mmol/mol)	39.2 ± 7.2	42.7 ± 14.4	33.2 ± 4.8	36 ± 9.3	34 ± 4.7	34.5 ± 8.2	34.3 ± 4.9	36.3 ± 11.5	0.293	<0.001	0.47
Glucose (mg/dL)	102.3 ± 16.4	112.9 ± 41.3	90.8 ± 8.1	98.9 ± 13.5	87.9 ± 9.1	89.2 ± 8.1	89.2 ± 11.2	90.6 ± 11.9	0.098	<0.001	0.341
Hemoglobin (g/dL)	13.8 ± 1.5	14.2 ± 1.8	13.6 ± 1.2	14.1 ± 1.3	13.8 ± 1.5	14.2 ± 1.2	13.7 ± 1.4	14.2 ± 1.3	0.271	0.726	0.907
HOMA-IR	5.6 ± 5.5	6.4 ± 6.4	2 ± 1	3.5 ± 2.7	1.7 ± 0.9	2.1 ± 1.4	1.6 ± 0.6	2.2 ± 2.2	0.167	<0.001	0.607

Abbreviations: Vit. D—vitamin D; Vit. B_12_—vitamin B_12_; LDH—lactate dehydrogenase; ALT—alanine aminotransferase; AST—aspartate aminotransferase; GGT—gamma-glutamyl transferase; ALP—alkaline phosphatase; TG—triglycerides; HDL—high-density lipoprotein; LDL—low-density lipoprotein; HbA1c%—glycated hemoglobin percentage; HbA1c—glycated hemoglobin; HOMA-IR—homeostatic model assessment for insulin resistance.

**Table 5:** Comparison of Clavien–Dindo classification outcomes between groups.

Clavien–Dindo Grade	Placebo *n* (%)	Probiotics *n* (%)	*p*-Value
all	2 (7.7)	3 (13.6)	0.649
I	0	1 (4.5)	0.458
II	2 (7.7)	0	0.493
IIIa	0	1 (4.5)	0.458
IIIb	0	1 (4.5)	0.458
IVa	0	0	1
IVb	0	0	1
V	0	0	1

Types of complications occurring according to the Clavien–Dindo classification: Grade I—vomiting; Grade II—abdominal pain and excessive drainage of the surgical wound; Grade IIIa—gastrointestinal bleeding; Grade IIIb—wound dehiscence accompanied by surgical site infection; Grade IV and V—no complications.

## Data Availability

The data sets used and/or analyzed during the current study are available from the corresponding author upon reasonable request as we are currently working on additional publications from this project, and some of the data will be used in these forthcoming studies.
